# 

**Published:** 1843-12-23

**Authors:** 


CONTENTS.

*Clinical Lectures and Reports.—Jefferson Medical College.*

Clinic of Professor Mütter.

Case 1. Tumour of the Nape of the Neck, 289

Case 2. Congenital Icthyosis, - - 289

Case 3. Lupus of the Lower Lid, - - 289

Case 4. Cancer of the Lip, - - - 290

Case 5. Cephalomatous Tumour, - - 290

Clinic of Dr. Pancoast.

Case of Paracentesis Cerebri, - - - 291

*Columbia College, D. C.*

Clinic of Dr. William P. Johnston.

Amaurosis, - - - - - - 292

Lenticular Cataract, (Hard,) - - 293

*Philadelphia Hospital.*

Service of M. Clymer, M. D.,

Case 1. Pneumonia — Bronchitis — Diarrhœa—Pregnancy—Recovery, - - 294

Case 2. Fever—Extensive Bronchitis, with absence of all rational signs. Intestinal Hemorrhagy—Recovery, - - - 294

Case 3. Typhoid Fever—Uterine Hemorrhage—Death—Autopsy—Remarks, - 296

*Bibliographical Notices.*

A Practical Treatise on the Diseases of Children. By D. Francis Condie, M. D., etc. etc., - - - - - - - 297

A Treatise on Dislocations and Fractures of the Joints. By Sir Astley Cooper, Bart., F. R. S., etc. A New Edition, much enlarged. Edited by Bransby B. Cooper, F.R.S., Surgeon to Guy's Hospital. With additional Observations, and a Memoir of the Author, - - - - - - 298

*Foreign Correspondence,* - - - 298

Dr. Craigie on the Pathological Causes of Cyanosis, - - - - - - 300

